# Automated Analysis of Pelvic Radiographs for Hip Dysplasia Screening Using Artificial Intelligence in Children with Cerebral Palsy: A Systematic Review

**DOI:** 10.3390/medicina62030570

**Published:** 2026-03-18

**Authors:** Ayesha Barmare, Erich Rutz, Sharmala Thuraisingam, Daniel Gould

**Affiliations:** 1 Department of Paediatrics, The Royal Children’s Hospital, The University of Melbourne, Melbourne 3052, Australia; ayesha.barmare@unimelb.edu.au (A.B.); sharmala.thuraisingam@unimelb.edu.au (S.T.); daniel.gould@unimelb.edu.au (D.G.); 2 Department of Paediatrics, Bob Dickens Chair, Paediatric Orthopaedic Surgery, The University of Melbourne, Melbourne 3010, Australia; 3 Department of Orthopaedics, The Royal Children’s Hospital, Melbourne 3052, Australia; 4 Murdoch Children’s Research Institute, Melbourne 3052, Australia; 5 Medical Faculty, University of Basel, 4001 Basel, Switzerland; 6 School of Health and Biomedical Sciences, Royal Melbourne Institute of Technology (RMIT University), Melbourne 3000, Australia

**Keywords:** cerebral palsy, artificial intelligence, hip dysplasia, musculoskeletal pathology, automated analysis

## Abstract

*Background and Objectives:* Cerebral palsy is a debilitating and complex movement disorder affecting millions of people worldwide. Many children with cerebral palsy develop hip dysplasia, which can lead to pain, functional decline, and long-term complications. Regular hip surveillance is therefore essential to allow early intervention and prevent progression. At present, screening is performed manually by experienced clinicians, which can be time consuming and costly. This study aimed to compare the performance of artificial intelligence models with expert clinicians in detecting hip dysplasia in children with cerebral palsy. *Materials and Methods:* A thorough search of Embase, Ovid MEDLINE, and Web of Science was conducted from inception to July 2025. Studies evaluating AI-based detection of hip dysplasia in children aged 18 years or younger with cerebral palsy were included. Risk of bias was assessed using the QUADAS-2 tool. Results were synthesised narratively in accordance with SWiM guidelines. *Results:* Across the six included studies, which included over 4000 radiographs, AI sensitivity for detecting hip dysplasia ranged from 70% to 97.4%, and specificity ranged from 85% to 96%, depending on the migration percentage thresholds applied. Area under the curve values ranged from 0.923 to 0.999. Only one study performed external validation using a national surveillance dataset. Risk of bias was moderate to high in most studies due to internal validation and small datasets. *Conclusions:* The findings suggest that AI demonstrates potential as an adjunct for hip surveillance in children with cerebral palsy.

## 1. Introduction

### 1.1. Background

Cerebral palsy (CP) is a group of permanent movement disorders in children caused by damage to the central nervous system (CNS) in an early stage of development. It is a leading cause of motor disability in children [1]. Hip dysplasia is a very common complication occurring in children with CP [2]. It is the result of the hip joint failing to grow in the correct way, causing an improperly formed joint to develop due to the femoral head fitting incongruently within the acetabulum [3,4]. This condition can be debilitating and early detection is necessary in order to adequately treat and prevent greater lasting consequences [5]. Routine surveillance, usually manually performed by experts, is important in the care of those with CP [6]. These manual measurements rely heavily on the expertise of the assessor and thus can lack efficiency. Artificial intelligence (AI) may represent a method of automating such measurements, thus increasing their efficiency and efficacy.

### 1.2. Existing Literature and Its Limitations

AI has become increasingly integrated into the healthcare system, with one of its most prominent applications being in the field of medical imaging [7]. In recent years, a growing number of studies have investigated the role of AI in detecting musculoskeletal conditions, using multiple different modalities such as X-ray, MRI, and ultrasound [8]. Most of this research has focused on adult populations, such as the detection of osteoarthritis or fractures [9], and some studies have evaluated the use of AI in the context of developmental dysplasia of the hip (DDH) in infants [10]. Despite this growing body of literature, there still appears to be a gap in the research. To the authors’ knowledge, no review has been published focusing specifically on the diagnostic performance of AI in detecting hip dysplasia in children with CP. Given that children with CP have a distinct anatomy, this can make the automation of screening for pathology more challenging and complex. Hence, it is necessary to conduct a systematic review investigating the use of AI tools in providing reliable, accurate, and clinically meaningful support in the radiographic surveillance of this high-risk population.

### 1.3. Objectives

This review aims to determine whether the use of AI can increase the efficiency and efficacy of hip dysplasia detection in children with CP compared to the manual measurements performed by experts.

## 2. Materials and Methods

### 2.1. Methods

This systematic review was performed in line with recommendations from the Preferred Reporting Items for Systematic Reviews and Meta-Analysis (PRISMA) statement guidelines and checklist (Supplementary Materials) [11]. A PICOTT (population, intervention, comparison, outcome, type of study and timeframe) framework was used to define the topic of interest [12]:

P—children (≤18 years old) with CP
I—hip dysplasia/displacement on radiographs detected using AI
C—hip dysplasia/displacement on radiographs detected by experts
O—sensitivity, specificity and accuracy
T—diagnostic accuracy studies
T—no restrictions to time of follow-up

### 2.2. Eligibility Criteria

- Children (0–18 years old) with CP
- Hip dysplasia/displacement detected on radiographs
- Human studies
- Diagnostic accuracy studies

### 2.3. Registration

The review protocol is publicly available on the PROSPERO database (CRD420251037304). The registered title was subsequently refined to more accurately reflect the scope and focus of the completed review.

### 2.4. Information Sources

The systematic search was performed on the following databases: Embase, Ovid Medline and Web of Science (WOS), in line with published recommendations from Bramer et al. [13]. The date the last search was performed was on the 19 July 2025. The reference lists of included studies were also screened for any relevant articles. 

### 2.5. Search Strategy

The search strategy was developed in conjunction with an experienced research librarian to ensure a comprehensive and reproducible search. Controlled vocabulary terms and free text keywords related to cerebral palsy, hip displacement, migration percentage, artificial intelligence, and machine learning were combined using Boolean operators. The strategy was then adapted for each database to maximise retrieval of relevant studies. A language limit was applied to include only articles published in English. Key words included cerebral palsy, hip dysplasia, hip dislocation, or hip displacement, artificial intelligence and machine learning. A detailed example of the search strategy is showcased in Table 1.

### 2.6. Data Collection Process

The search files were imported into Covidence to delete duplicates and organise the studies for screening. Two reviewers independently selected the studies based on title and abstract, and in a second phase read the studies in full to obtain the final number of studies included. After selection, the data of first author, year of publication, population and participant characteristics was collected, as well as the main outcomes. The data was then extracted and recorded on an Excel template by two authors. Any disagreements were resolved through discussion.

### 2.7. Data Items

The outcomes of interest were performance metrics including sensitivity, specificity, accuracy, precision, recall, mean absolute error (MAE), area under the receiver operating characteristic curve (AUC), and intraclass correlation coefficient (ICC). Other variables included study design, sample size, imaging modality, AI model type, task performed, reference standard, and validation method.

### 2.8. Risk of Bias Assessment

Risk of bias for diagnostic accuracy studies was assessed using the QUADAS 2 tool. One reviewer assessed each study using the QUADAS 2 tool and the second reviewer assessed two studies in order to compare QUADAS 2 outcomes and to ensure a streamlined process. Prior to undertaking the assessment, both reviewers discussed and agreed upon a predefined set of grading criteria to standardise interpretation of the QUADAS 2 domains. This included establishing clear expectations for what would constitute low, moderate, and high risk of bias within each domain to ensure a consistent evaluation process. Any uncertainties or discrepancies arising during the assessment were resolved through discussion and consensus between the reviewers. The assessments are summarised in Table 2. The most common sources of bias include non-consecutive patient selection and no pre-specified diagnostic thresholds. The QUADAS 2 tool was selected as it provides a simple and intuitive method to clearly critically appraise the included studies in this review. With 4 key domains being included, this tool allowed for a clear-cut method to evaluate the quality of the reviewed literature [14]. The potential for reporting bias was considered qualitatively by reviewing study protocols and comparing outcomes with stated objectives; however, no formal statistical assessment of reporting bias was conducted.

### 2.9. Synthesis Methods

As no meta-analysis was performed, heterogeneity was assessed qualitatively by comparing study design, dataset size, AI model type, outcome thresholds, and performance metrics across studies. No sensitivity analysis was conducted. Findings of studies were synthesised using a structured narrative synthesis approach in accordance with the SWiM (Synthesis Without Meta-analysis) [21] guidelines. Studies were grouped by outcome measures, with results reported descriptively using extracted performance metrics (e.g., sensitivity and specificity), allowing qualitative comparison of outcomes across studies without statistical aggregation. Performance metrics were extracted as reported in the original studies, including sensitivity, specificity, accuracy, AUC, ICC, MAE, precision, and recall. No imputation of missing statistics or data conversion was undertaken. Furthermore, the significant heterogeneity in study outcomes, the lack of consistently reported confidence intervals in most included studies, and the absence of a meta-analysis being conducted meant that the certainty of evidence was not formally assessed.

## 3. Results

### 3.1. Study Selection

**Figure 1:**
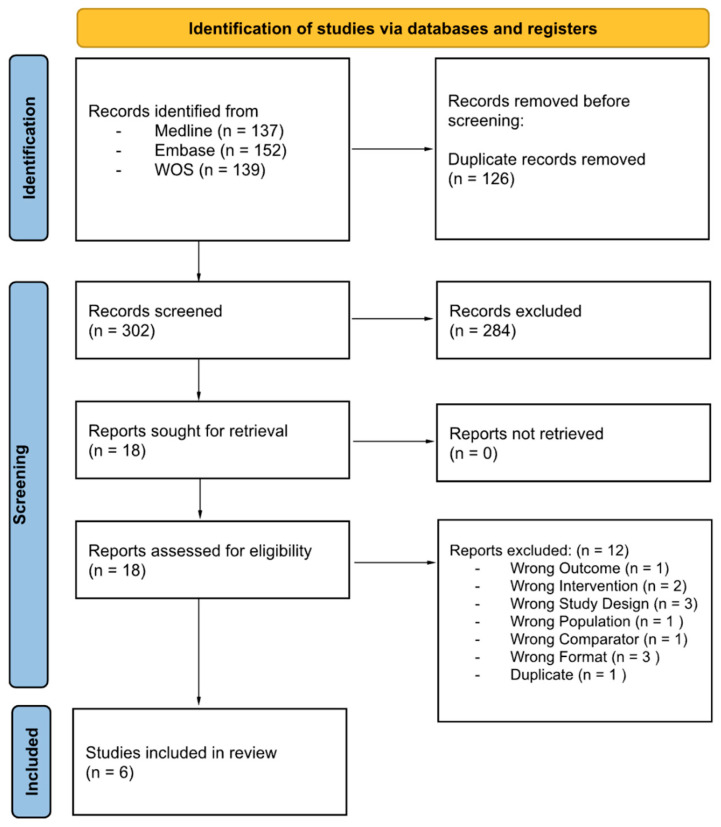
Study selection flowchart.

The search returned 428 studies initially, and ultimately six studies remained for inclusion into this systematic review. Initially, titles and abstracts were screened against predefined inclusion and exclusion criteria. Then, full text articles were reviewed to confirm eligibility. Any disagreements between reviewers were resolved through discussion and consensus. The study selection process is shown in Figure 1.

### 3.2. Study Characteristics

Characteristics of included studies have been summarised below in Table 3. The studies were published between 2021 and 2025 and included sample sizes ranging from 122 to 1650 radiographs. All studies involved paediatric patients with CP, ages 1 to 18 years, however only a select few studies reported the Gross Motor Function Classification System (GMFCS) levels or CP subtypes. All included studies used anteroposterior pelvic (AP) radiographs as the imaging modality and a variety of AI model types were included such as machine learning (ML), convolutional neural networks (CNNs), and other deep learning (DL) approaches. The typical reference standard used was human raters using MP thresholds.

Study characteristics are summarised in Table 3.

### 3.3. Study Biases

The most common sources of bias within the studies included were non-consecutive patient selection and no pre-specified diagnostic thresholds.

### 3.4. Diagnostic Accuracy

From the six studies included in this review, the sensitivity of AI screening of hip dysplasia ranged from 70% to 97.4%, and specificity from 85% to 96%, depending on MP thresholds used. MP threshold values ranged from <30% to ≥50%. The AUC values were 0.923 (Yeh et al.) [20], up to 0.999 for dislocated hips (Ertan Birsel et al.) [15]. In Hughes et al. [16], the AUC for detecting hip displacement was 0.95, 0.97, and 0.98 at MP thresholds of ≥30%, ≥40%, and ≥50%, respectively as highlighted in Table 4.

### 3.5. Model Generalisability/Validation

There was only one study which performed external validation using a national dataset (CPIPS) (Hughes et al.) [16] whilst the other five studies used internal validation (cross-validation or split samples). External validation is crucial in the evaluation of AI based diagnostic tools, as it provides evidence that a model can perform reliably beyond the dataset on which it was originally developed [22]. In medical imaging applications, model performance can be influenced by variations in patient populations, imaging equipment, and image quality across institutions. As a result, models that perform well on internal datasets may demonstrate reduced accuracy when applied to external datasets. Evaluating AI systems on independent datasets from different centres or surveillance programmes therefore plays an important part in assessing generalisability and ensuring that models are not overly tailored to the conditions under which they were originally trained [23].

### 3.6. AI vs Human Performance

All six of the studies reported comparable performance of the AI models vs expert raters in terms of measurements. AI was also shown to be more consistent especially when junior raters were involved or when MP thresholds were ambiguous. One study (Ertan Birsel et al.) [15] highlighted that the SVM model was able to resolve human disagreement cases with high accuracy (92.9%).

## 4. Discussion

### 4.1. Summary of Evidence

In general, these studies show that AI is able to match human rater measurement in detecting hip dysplasia in children with cerebral palsy. Overall, in all studies there were moderate to good ICC values and low MAE, suggesting a strong correlation between AI and manual measurement with respect to migration percentage (MP) estimation. In general, ICC values between 0.50 and 0.75 indicate moderate reliability, whilst values between 0.75 and 0.90 represent good reliability [24]. In the study by Hughes et al., ICC values ranged from 0.60 to 0.71, which fall within the moderate reliability range which is comparatively lower than the other studies within this review. This may be explained by the use of a large national surveillance dataset (CPIPS), which includes images acquired across multiple centres with varying radiographic quality and patient characteristics. Such heterogeneity likely introduces greater variability compared with smaller single centre datasets. It was also evident that inputs such as landmark detection often saw better outcome measures when compared to raw image inputs perhaps due to clearer anatomy. In most studies, human-derived measurements were used as inputs and training datasets for the AI models. Whilst expert annotation is the current clinical standard for hip surveillance, this approach introduces the possibility of circularity bias, as AI models are effectively trained to replicate human measurements and may therefore inherit the variability and subjectivity associated with manual assessment. As a consequence, these models are agreeing with expert raters rather than validating against an independent clinical reference standard. Furthermore, none of the included studies compared AI measurements with clinical outcomes, or alternative imaging modalities such as computed tomography (CT). Overall, this introduces the concern that the AI derived outputs may not be fully independent of the data on which they were trained [25].

### 4.2. Contribution to the Literature

This systematic review is the first to evaluate the use of AI for detecting hip dysplasia in children with CP It provides a detailed synthesis of AI model types, input features, and diagnostic outputs across multiple studies. By comparing AI performance to expert measurements, the review highlights the potential for integrating automated tools into hip surveillance for this vulnerable population. These findings can inform the development of more efficient, reliable AI systems to support clinicians involved in paediatric orthopaedic care.

### 4.3. Strengths and Limitations

Strengths of this study include its adherence to PRISMA guidelines [11], ensuring systematic reporting. To the authors’ knowledge, this is the first systematic review specifically evaluating the diagnostic performance of AI in detecting hip dysplasia in children with CP. A structured narrative synthesis was conducted in accordance with SWiM guidelines [21] and in addition to this, the use of the QUADAS-2 tool [14] strengthened the review through a formal and systematic assessment of risk of bias.

There were a number of limitations to this review. Firstly, given this study was limited to English language publications, this may mean that relevant non-English language studies were excluded. Additionally, grey literature and conference abstracts were intentionally excluded in order to support the methodological reproducibility of the review findings; this, however, means that other potentially relevant information was not synthesised. A further limitation is the relatively small number of eligible studies identified in the literature. This likely reflects the emerging nature of research exploring AI applications in hip surveillance for children with CP and underscores the need for larger, multicentre studies with robust external validation. Lastly, a meta-analysis was not performed on this dataset due to the heterogeneity in the studies, particularly involving model types and outcome reporting.

### 4.4. Clinical Implications

Clinically, automating the analysis of pelvic radiographs may assist healthcare professionals by providing rapid and reproducible measurements of hip dysplasia. This would be particularly valuable in large hip surveillance programmes where repeated radiographic assessments are required over time. AI-assisted tools may also help reduce inter-observer variability and support clinicians with less experience in interpreting paediatric pelvic radiographs.

### 4.5. Implications for Future Research

Due to the limited number of studies within this field, AI systems may not be able to replace the expertise of human raters, however they can serve as a reliable adjunct to the current systems in place. On a larger scale, for future development, it would be beneficial for paediatric orthopaedic bodies to develop guidelines for AI integration systems for better assimilation of these techniques into current practice. If future research were to be conducted in this area of study, external validation should be prioritised to verify the reproducibility of model performance across a large population. Efforts should also be made to minimise circularity bias, which can occur when AI systems are trained and evaluated using human-derived measurements. To reduce this risk, future studies should use independent datasets for training and testing, ensure clear separation between development and validation cohorts, and where possible compare AI outputs with objective imaging references such as computed tomography (CT) or other imaging modalities. There also needs to be a consistency in the reporting of performance metrics such as AUC and ICC, thus allowing for more consistent comparative analysis between studies.

## 5. Conclusions

Overall, the literature demonstrates that there is a place for the use and integration of AI in hip surveillance for children with CP. Whilst this may not be ready for independent use, it could be used as a reliable accessory to the current care provided. AI has the potential to reduce clinician workload, increase efficiency of results and improve as well as streamline access to screening, especially in environments where resources are limited.

## Figures and Tables

**Table 1:** Search strategy.

Database	Database String
Ovid Medline	exp Cerebral Palsy/ (cerebral palsy or diplegia or hemiplegia or quadriplegia or “little disease” or “little’s disease” or “littles disease”).mp. 1 or 2 exp Artificial Intelligence/or exp Machine Learning/or exp Deep Learning/or exp Neural Networks, Computer/or exp Pattern Recognition, Automated/or exp Image Processing, Computer-Assisted/ (“artificial intelligence” or AI or “machine learning” or “natural language processing” or “deep learning” or “computer vision” or “neural network”).mp. 4 or 5 exp Hip Dislocation/ (hip or hips).mp. 7 or 8 3 and 6 and 9
Embase	exp cerebral palsy/ (cerebral palsy or diplegia or hemiplegia or quadriplegia or “little disease” or “little’s disease” or “littles disease”).tw,kw,de. 1 or 2 exp artificial intelligence/or exp machine learning/or exp deep learning/or exp neural network/or exp pattern recognition/or exp image processing/ (“artificial intelligence” or AI or “machine learning” or “natural language processing” or “deep learning” or “neural network” or “computer vision”).tw,kw,de. 4 or 5 exp hip dislocation/ (hip or hips).tw,kw,de. 7 or 8 3 and 6 and 9
Web Of Science	TS = (“cerebral palsy” OR diplegia OR hemiplegia OR quadriplegia OR “little disease” OR “little’s disease” OR “littles disease”) AND TS = (“artificial intelligence” OR AI OR “machine learning” OR “natural language processing” OR “deep learning” OR “neural network” OR “computer vision”) AND TS = (“hip dislocation” OR “hip dysplasia” OR “hip displacement” OR hip OR hips)

**Table 2:** Risk of bias.

First Author	Patient Selection—Data	Patient Selection—RoB ^a^	Index Test—Data	Index Test—RoB ^a^	Reference Standard—Data	Reference Standard—RoB ^a^	Flow and Timing—Data	Flow and Timing—RoB ^a^	^b^ QUADAS-2 Comments
Ertan Birsel [15]	Two centres	Low	Cross-validation only; incorporation bias risk (human MP **^d^** features)	High	Resident + surgeon	Unclear	All hips analysed	Low	High risk due to size and incorporation bias risk, blinding unclear
Hughes [16]	External validation on national CPIPS **^c^** dataset	Low	Predefined thresholds; external validation	Low	Multiple clinicians	Low	All hips analysed	Low	Low risk across domains; strongest evidence base
Lam [17]	Single-centre	Unclear	Model selected post hoc; no external validation	Low	HipScreen manual by fellows	Unclear	All images analysed	Low	High risk for patient selection and index test
Pham [18]	Single-centre	Unclear	Thresholds retrospective; no external validation	Unclear	Expert ground truth; novice comparator	Unclear	All images analysed	Low	High risk in patient selection and index test
Thompson [19]	Single-centre	Unclear	No external validation; same-site testing	Low	Five experts	Unclear	All hips analysed	High	High risk in patient selection and index test; strong reference standard
Yeh [20]	Single-centre	Unclear	Optimised threshold at 27%; no external validation	High	Expert labels	Unclear	All images analysed	Low	High risk in patient selection and index test; blinding unclear

**^a^** Risk of bias. **^b^** Quality assessment of diagnostic accuracy studies, Version 2. **^c^** Cerebral Palsy Integrated Pathway Scotland. **^d^** Migration percentage.

**Table 3:** Study characteristics.

First Author	Study Title	Population Description (e.g., CP ^b^ Type, GMFCS ^c^ Level)	Age Range/Mean Age	Sample Size (Total)	AI ^a^ Model Type (e.g., ML ^d^, CNN ^g^, Deep Learning, Landmark Based)	Output (e.g., MP ^e^, HSA)
Ertan Birsel [15]	Machine learning-assisted classification of hip conditions in pediatric cerebral palsy patients using migration percentage measurements	CP ^**b**^ surveillance cohort	Not stated	88 radiographs (176 hips)	ML ^**d**^ (Support Vector Machine model)	3 class classification MP ^**e**^ values <30%—0. MP ^**e**^ values ≥30% and <60%—1. MP ^**e**^ values ≥60%—2.
Hughes [16]	Fully automated measurement of paediatric cerebral palsy pelvic radiographs with BoneFinder	CP ^**b**^ children; GMFCS ^**c**^ I–V	1–17 years	509 radiographs (1018 hips)	Landmark-based (BoneFinder)	Numeric measurement (MP ^**e**^ and HSA ^**f**^)
Lam [17]	An automated framework for pediatric hip surveillance and severity assessment using radiographs	CP ^**b**^ children	3–8 years	541 radiographs	Deep learning (CNN ^**g**^)	Numeric MP ^**e**^ measurement
Pham [18]	Assessment of hip displacement in children with cerebral palsy using machine learning approach	CP ^**b**^ children; GMFCS ^**c**^ II–V	4–10 years	122 radiographs	Deep learning (CNN ^**g**^)	Numeric MP ^**e**^ measurement
Thompson [19]	Automating Radiographic Measurements of the Hip in Children with Cerebral Palsy	CP ^**b**^ children	Mean ~8.3 years	1650 radiographs	Landmark-based (BoneFinder)	Numeric MP ^**e**^ measurement
Yeh [20]	Automated Measurement of Migration Percentage in Hip Surveillance Radiographs: Development and Testing of a Deep-Learning AI Algorithm	CP ^**b**^ children; GMFCS ^**c**^ I–V	2–18 years	1275 radiographs	Deep learning (ResNet)	Numeric MP ^**e**^ measurement

**^a^** Artificial intelligence. **^b^** Cerebral palsy. **^c^** Gross motor function classification system. **^d^** Machine learning. **^e^** Migration percentage. **^f^** Head shaft angle. **^g^** Convolutional neural network.

**Table 4:** Results of individual studies.

First Author	Performance Metric 1 (e.g., Precision, Sensitivity and Specificity)	Performance Metric 2 (e.g., AUC ^a^, ICC ^b^, Accuracy, MAE ^c^)	Inter-Rater Reliability (If Reported)
Ertan Birsel [15]	Average precision 0.93 Sensitivity 92.898% and Specificity 96.449%	Accuracy ~92.9% AUC ^**a**^ up to 0.999 for dislocated hips	Human–human ICC ^**b**^ 0.951–0.976
Hughes [16]	Sensitivity and specificity were highlighted in graphs visually however were not reported as exact numerical values in the original study and therefore could not be directly extracted.	ICC ^**b**^ of MP ^**d**^—0.60–0.71 ICC ^**b**^ of HSA ^**e**^—0.60–0.64 AUC ^**a**^ 0.95/0.97/0.98 for MP ^**d**^ ≥ 30/40/50%	Human–human ICC ^**b**^ > 0.90
Lam [17]	Average precision 95.64% Average recall 92.42%	Best MAE ^**c**^ ~0.049	Not reported
Pham [18]	MP ^**d**^ ≤ 30% and >30%—Sensitivity 87.8%, Specificity 93.4% MP > 40%—Sensitivity 63.2%, Specificity 94.5%.	Accuracy 90.9% for MP ^**d**^ >30% and MP ^**d**^ ≤ 30% ICC ^**b**^ of MP ^**d**^—0.91	Human–human ICC ^**b**^ 0.92
Thompson [19]	MP ^**d**^ ≥ 30%—Sensitivity 91.4–92.0%, Specificity 86.6–86.8% MP ^**d**^ ≥ 40%—Sensitivity 90.9–92.0%, Specificity 94.1–95.0% MP ^**d**^ ≥ 50%—Sensitivity 90.5–93.4%, Specificity 97.3–97.7%	MP ^**d**^ ≥30%—Accuracy 88.7% MP ^**d**^ ≥40%—Accuracy 93.4–94.2% MP ^**d**^ ≥50%—Accuracy 96.1–97.0% ICC ^**b**^ of MP ^**d**^—0.91 ICC ^**b**^ of HAS ^**e**^ 0.73	Not reported
Yeh [20]	MP ^**d**^ > 30%—Sensitivity 70%, Specificity 94% MP ≥ 27%—Sensitivity 85%, Specificity 85%	AUC ^**a**^ 0.923	Not reported

**^a^** Area under the curve. **^b^** Intraclass correlation coefficient. **^c^** Mean absolute error. **^d^** Migration percentage. **^e^** Head shaft angle.

## Data Availability

No new data were created or analysed in this study. Data supporting the findings of this study are available within the cited published articles.

## Supplementary Materials

The following supporting information can be downloaded at: https://www.mdpi.com/article/10.3390/medicina62030570/s1, PRISMA 2020 Checklist [11].

## Abbreviations

The following abbreviations are used in this manuscript:

AI	Artificial Intelligence
AP	Anteroposterior
AUC	Area Under the Curve
CNN	Convolutional Neural Network
CP	Cerebral Palsy
CPIPS	Cerebral Palsy Integrated Pathway Scotland
CNS	Central Nervous System
DDH	Developmental Dysplasia of the Hip
GMFCS	Gross Motor Function Classification System
HSA	Head Shaft Angle
ICC	Intraclass Correlation Coefficient
MAE	Mean Absolute Error
MP	Migration Percentage
NSA	Neck Shaft Angle
PICOTT	Population, Intervention, Comparison, Outcome, Type of Study and Timeframe
PRISMA	Preferred Reporting Items for Systematic Reviews and Meta-Analyses
RoB	Risk of Bias
SVM	Support Vector Machine
SWiM	Synthesis Without Meta-analysis
WOS	Web of Science
